# Chemical Composition Analysis, Antimicrobial Activity and Cytotoxicity Screening of Moss Extracts (Moss Phytochemistry)

**DOI:** 10.3390/molecules200917221

**Published:** 2015-09-18

**Authors:** Laura Klavina, Gunta Springe, Vizma Nikolajeva, Illia Martsinkevich, Ilva Nakurte, Diana Dzabijeva, Iveta Steinberga

**Affiliations:** 1 Department of Environmental Science, University of Latvia, 19 Raina Blvd., Riga LV-1586, Latvia; E-Mail: iveta.steinberga@lu.lv; 2 Institute of Biology, University of Latvia, 3 Miera Street, Salaspils LV-2169, Latvia; E-Mail: gunta.springe@lu.lv; 3 Department of Microbiology and Biotechnology, University of Latvia, 4 Kronvalda Blvd., Riga LV-1010, Latvia; E-Mail: vizma.nikolajeva@lu.lv; 4 Faculty of Chemistry, University of Latvia, 19 Raina Blvd., Riga LV-1586, Latvia; E-Mails: martsinkevich.illia@gmail.com (I.M.); ilva.nakurte@lu.lv (I.N.); diana.dzabijeva@lu.lv (D.D.)

**Keywords:** extraction, antiradical activity, GC/MS, LC-TOF-MS, cytotoxicity, antimicrobial activity, polyphenols, amino acids

## Abstract

Mosses have been neglected as a study subject for a long time. Recent research shows that mosses contain remarkable and unique substances with high biological activity. The aim of this study, accordingly, was to analyze the composition of mosses and to screen their antimicrobial and anticancer activity. The total concentration of polyphenols and carbohydrates, the amount of dry residue and the radical scavenging activity were determined for a preliminary evaluation of the chemical composition of moss extracts. In order to analyze and identify the substances present in mosses, two types of extrahents (chloroform, ethanol) and the GC/MS and LC-TOF-MS methods were used. The antimicrobial activity was tested on four bacteria strains, and the anticancer activity on six cancer cell lines. The obtained results show the presence of a high number of primary (fatty acids and amino acids), but mainly secondary metabolites in moss extracts—including, sterols, terpenoids, polyphenols and others—and a high activity with respect to the studied test organisms.

## 1. Introduction

Mosses belong to the simplest land plants. At the same time, they belong to the second largest taxonomic group in the plant kingdom: bryophytes [1]. There are around 25,000 bryophyte species, which can be found in most ecosystems worldwide and include mosses (*Musci* ~ 8000 species), liverworts (*Hepaticae* ~ 6000 species) and hornworts (*Anthocerotae* ~ 1000 species). Due to their small size and identification problems, bryophytes have for a long time been neglected and considered almost useless as a source of biologically-active substances. Now, the situation has changed, and the interest in bryophyte chemical composition is increasingly growing, as a high number of biologically-active compounds recently have been found in mosses and liverworts [1,2,3]. Many compounds that have been isolated from bryophytes have shown high biological activity [3,4,5]. Therefore, extracts of bryophytes are prospects for the search of new pharmaceutically-active compounds [6,7]. Well-expressed antibacterial, antifungal and antiviral activities have been demonstrated in a number of bryophytes, and their cytotoxicity with respect to cancer cells, their antioxidant, antiplatelet, antithrombin, insecticidal and neuroprotective activity, as well as the ability to inhibit a number of biochemically important enzymes, as well as other kinds of activities, have been confirmed in several studies [3,4,7,8]. For all that, from a large number of bryophytes, only a negligible number of species has been extensively studied, and most of the recent studies have concentrated on liverwort composition [1], especially on the substances present in the oil bodies of these plants.

The largest taxonomical group of bryophytes is mosses. Mosses are an important element of ecosystems, especially in the Northern Hemisphere, where they are the main peat-forming plants (*Sphagnum mosses*). They are also important in forest ecosystems. Traditionally, mosses and their extracts have found application in ethnopharmacology in northern countries, in Siberia and elsewhere. North American tribes used mosses as medical plants for the treatment of wounds and burns and for curing tuberculosis, pneumonia, neurasthenia and other illnesses [8,9]. Mosses have a well-known antimicrobial activity [10,11].

The major structural components of mosses are carbohydrates [12,13], and they also contain secondary metabolites with possibly high biological activity [1]. To date, the composition of mosses has not been much studied from the perspective of their application potential, but mainly has been concentrated on the investigation of specific substance groups, such as fatty acids, lipids, essential oils *etc.* [14,15]. Another reason to study the composition of bryophytes is related to the need to understand their metabolism. It is important to study secondary metabolites in mosses, as they are different from those in higher plants [16]. Studies of secondary metabolites can help to understand the stress reactions (drought/wetness), oxidative stress, pollution stress (for example, heavy metal impact) and UV radiation impact reaction in bryophytes, as well as the functions of the main secondary metabolites in the overall metabolism [16,17,18,19]. To advance the understanding of bryophyte chemical composition, we performed chromatographic analysis of bryophyte extracts and the relation of these to biological activity.

The aim of the study was to analyze the composition of moss extracts by means of GC/MS and LC-TOF-MS and to screen their antimicrobial and anticancer activity.

## 2. Results and Discussion

In the search for new biologically-active compounds, mosses can be considered as a largely neglected material, as there have been much fewer studies dedicated to the composition of secondary metabolites and their biological activity in mosses compared to, for example, similar studies of higher plants [1]. This is especially the case with respect to the studies of moss composition, notwithstanding the large number of known species. In this study, the mosses common to the natural environment of Northern Europe were selected. The mosses were sampled in forest, wetland and bog territories. They are abundant in nature (Table 1) and can also be easily cultivated [20].

**Table 1:** Studied moss species with the corresponding codes and growth conditions.

Species	Code	Growth Conditions
*Aulacomnium palustre*	AP	Edges of bogs and humid areas; forms homogenous coverage of moss
*Climacium dendroides*	CD	Deciduous forest, humid, shadowy areas
*Dicranum polysetum*	DP	Deciduous forests
*Hylocomium splendens*	HS	Deciduous forests with poor soil; commonly found together with *Pleurozium schreberi*
*Pleurozium schreberi*	PS	Deciduous and coniferous forests, heaths with poor soil; commonly found together with *Hylocomium splendens*
*Polytrichum commune*	PC	Coniferous forests, humid habitats; sometimes found together with *Sphagnum girgensohnii*
*Polytrichum juniperum*	PJ	Coniferous forests, raised and transitional bogs, near decaying wood or tree bases
*Ptilium crista-castrensis*	PCC	Mixed forests, sunny and moderately humid areas
*Rhytidiadelphus triquetrus*	RT	Mixed forests, near tree bases
*Sphagnum fallax*	SF	Middle of bogs, bog pools
*Sphagnum magellanicum*	SM	Bogs with acidic soil, bog pools or near them
*Sphagnum rubellum*	SR	Middle of bogs, bog pools and other very humid areas
*Sphagnum tenellum*	ST	Edges of bogs, bogs, humid areas

Mosses are composed of polysaccharides of different compositions [13]. Moss secondary metabolites are lower polarity substances (lipids) and polar substances (carbohydrates, polyphenols and others). For the isolation of major groups of secondary metabolites, a sequential extraction approach can be used, isolating lipids initially and more polar substances afterwards [21]. Considering the experience in studies of higher vegetation [22], two solvents were selected for the extraction of secondary metabolites from mosses: (1) chloroform; (2) ethanol (Table 2).

The studied mosses contain relatively high concentrations of secondary metabolites (Table 2), and the amount of more polar substances (in ethanol extracts) is lower than that of lipids (chloroform extracts). Mosses contain high concentrations of polyphenols (from 200 mg up to 800 mg gallic acid equivalent/100 g of dry weight), whereas their radical scavenging activity (estimated as the inhibition of 2.2-diphenyl-1-picrylhydrazyl radical) is much less than that in higher plants [23]. In higher plants, polyphenolic substances determine the radical scavenging activity. In bryophyte extracts, in contrast, there are no correlations (correlation coefficient < 0.3) between the total polyphenol concentration and the radical scavenging activity (Table 2).

**Table 2:** Total polyphenolics, total carbohydrates and radical scavenging activity of ethanol extracts * of the studied mosses and the yields of chloroform extracts ** expressed as mg per 100 g of dry moss weight.

Species Code	Total Polyphenol Content, GE	Total Carbohydrates, GLE	Radical Scavenging Activity, GE	Extraction Yield *, mg	Extraction Yield **, mg
**AP**	267.5 ± 13.1	10.6 ± 0.4	39.0 ± 1.9	114.2 ± 5.7	259 ± 12.8
**CD**	272.1 ± 14.3	7.6 ± 0.3	42.3 ± 2.8	98.6 ± 4.7	412 ± 22.7
**DP**	399.2 ± 19.5	8.4 ± 0.3	16.4 ± 0.8	133.5 ± 6.7	482 ± 24.2
**HS**	298.4 ± 14.6	6.9 ± 0.3	59.3 ± 3.1	102.6 ± 5.1	443 ± 23.2
**PS**	275.6 ± 13.4	11.4 ± 0.5	15.6 ± 0.8	145.2 ± 7.3	561 ± 27.9
**PC**	800.7 ± 39.1	12.1 ± 0.5	12.6 ± 0.6	154.8 ± 7.7	513 ± 26.1
**PJ**	416.2 ± 20.2	5.6 ± 0.2	24.1 ± 1.2	88.5 ± 4.4	510 ± 26.1
**PCC**	237.9 ± 11.7	9.7 ± 0.3	19.6 ± 1.0	145.5 ± 7.3	464 ± 23.9
**RT**	379.1 ± 18.5	15.3 ± 0.7	11.3 ± 0.5	144.8 ± 7.2	451 ± 23.5
**SF**	783.2 ± 38.1	8.9 ± 0.4	17.6 ± 0.9	142.5 ± 7.1	213 ± 10.1
**SM**	370.6 ± 18.1	6.6 ± 0.3	18.2 ± 0.9	140.6 ± 7.0	378 ± 18.4
**SR**	345.7 ± 16.7	10.3 ± 0.4	16.3 ± 0.8	65.3 ± 3.3	181 ± 9.8
**ST**	280.1 ± 13.7	7.9 ± 0.3	14.9 ± 0.7	149.3 ± 7.4	240 ± 11.6

### 2.1. GC/MS Analysis of the Moss Chloroform Extract

**Figure 1:**
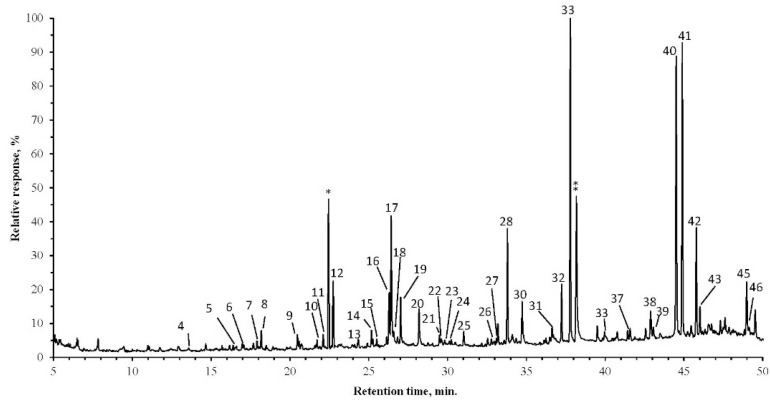
Gas chromatogram with mass-spectrometric detection of the total lipid extract of *Polytrichum commune* (PC). IS, internal standard (* methylheptadecanoic acid, ** progesterone); numbers refer to the compounds listed in Table 1. Acid and alcohol groups were derivatized prior to the GC/MS analysis.

**Figure 2:**
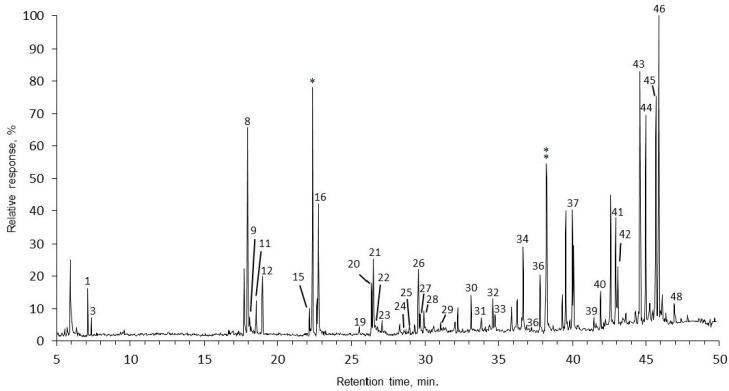
Gas chromatogram with mass-spectrometric detection of the total lipid extract of *Rhytidiadelphus triquetrus* (RT). IS, internal standard (* methylheptadecanoic acid, ** progesterone); numbers refer to the compounds listed in Table 1. Acid and alcohol groups were derivatized prior to the GC/MS analysis.

Moss lipids have been an object of pioneering studies [24] of bryophyte secondary metabolites. In biogeochemistry and paleoclimatology, studies have focused on the presence of alkanes in mosses [25]. However, not much attention has been paid to lipids recently. Chromatographic analysis reveals significant differences in the lipid fraction composition depending on the moss species (Figure 1 and Figure 2).

The analysis of freely available lipids (Table 3 and Table 4) reveals a high number of different groups of substances (altogether, 88 different substances have been identified and quantified in the studied mosses) playing significant functions in the moss metabolism and possibly affecting the biological activity of their extracts. There were significant differences among the studied moss species: Many substances have been quantified only in some moss species, and this is first of all relevant with respect to the most biologically-active substances, like sterols and terpenoids. So, high concentrations of ergost-7-en-3-ol and pimaric acid were found in *Ptilium crista-castrensis*, while other sterols were found in comparatively higher concentrations in *Polytrichum commune* than in other mosses. The highest number of substances was found in *Pleurozium schreberi* and the lowest in *Sphagnum magellanicum*. The following substances have been identified in mosses for the first time: diploptene, α-amyrin, oleanolic acid, uvaol, cycloartenol, ursolic acid, neophytadiene, and others. The studied mosses contain a significant number of alkanes and fatty acids, including many unsaturated fatty acids and fatty alcohols. With respect to the functions of secondary metabolites, the presence of sterols and terpenoids can be considered as important.

Both Figure 1 and Figure 2 show the differences in the extract GC/MS chromatograms depending on the moss species. Not only the substances, but also the quantities of the identified substances were evidently different. Some substances, such as succinic, malic and isopimaric acids, as well as diploptene, cycloartenol, and others, could be found only in one moss extract. Although most of the substances were identified and quantified, some of them could not be identified by their mass spectra. Identification of unknown substances could be achieved by fractionation; therefore, substance concentration and purification could aid their identification.

In order to better understand the chemical composition of mosses, a total of 13 moss samples was analyzed. The GC/MS data of the analyzed mosses reveal a high variability of substances and their quantities (Table 4).

**Table 3:** Concentrations (mg/100 g dry weight) of substances in the *Polytrichum commune* and *Rhytidiadelphus triquetrus* chloroform extracts according to the GC/MS analysis.

No. *	RT **	RI_C_ ***	Components	*Rhytidiadelphus triquetrus*	*Polytrichum commune*
1.	7.11	1289	2,4-Decadienal	19.0 ± 0.5	2.5 ± 0.5
2.	7.14	1306	Succinic acid	0.8 ± 0.1	ND
3.	7.37	1351	Nonanoic acid	7.4 ± 0.2	1.2 ± 0.2
5.	10.03	1481	Malic acid	0.6 ± 0.2	ND
6.	13.59	1645	Dodecanoic acid	0.8 ± 0.1	3.3 ± 0.1
7.	16.42	1784	2-Methylfructofuranoside	0.6 ± 0.1	2.8 ± 0.1
8.	16.97	1811	Azelaic acid	0.3 ± 0.1	3.5 ± 0.1
9.	17.96	1844	Neophytadiene	123.9 ± 1.5	5.6 ± 0.2
10.	18.09	1846	Hexahydrofarnesyl acetone	5.3 ± 0.2	1.3 ± 0.1
11.	18.20	1852	Tetradecanoic acid	2.7 ± 0.2	11.1 ± 0.2
12.	18.53	1862	Phyta-1,3(*Z*)-diene	16.0 ± 0.5	1.7 ± 0.1
13.	18.97	1877	Phyta-1,3(*E*)-diene	34.4 ± 0.5	1.2 ± 0.1
14.	20.47	1940	Pentadecanoic acid	0.3 ± 0.1	8.7 ± 0.5
15.	21.73	2000	Eicosane	0.5 ± 0.1	5.6 ± 0.2
16.	22.20	2009	9-Hexadecenoate + 7,10,13-Hexadecatrienoic acid	4.6 ± 0.3	8.9 ± 0.3
17.	22.77	2040	Hexadecanoic acid	68.3 ± 1.7	2.6 ± 0.2
18.	24.38	2109	9-Heptadecenoic acid	0.4 ± 0.1	6.4 ± 0.2
19.	25.17	2149	1-Octadecanol	0.4 ± 0.1	8.6 ± 0.7
20.	25.56	2165	Phytol	12.6 ± 0.6	5.3 ± 0.3
21.	26.33	2201	9,12-Octadecadienoic acid (*Z*,*Z*)-,	28.3 ± 1.6	31.2 ± 1.6
22.	26.49	2207–2209	9-Octadecenoic acid + (3*Z*,6*Z*,9*Z*)-3,6,9-Octadecatrienoate	46.7 ± 2.7	74.2 ± 3.2
23.	26.62	2214	11-Octadecenoic acid	3.2 ± 0.4	11.1 ± 0.5
24.	27.06	2235	Octadecanoic acid	6.6 ± 0.3	25.5 ± 0.8
25.	28.56	2295	Pimaric acid + C23	3.3 ± 0.2	27.8 ± 1.6
26.	28.92	2326	Isopimaric acid	6.2 ± 1.1	ND
27.	29.54	2355	*cis*-5,8,11,14-Eicosatetraenoic acid	30.6 ± 1.3	7.5 ± 0.5
28.	29.64	2361	*cis*-5,8,11,14,17-Eicosapentaenoic acid	8.8 ± 0.8	5.5 ± 0.3
29.	29.93	2369	Dehydroabietic acid	18.4 ± 0.2	4.8 ± 0.4
31.	31.06	2432	Eicosanoic acid	6.0 ± 0.3	7.9 ± 1.2
32.	33.13	2546	1-Docosanol	19.9 ± 1.3	28.7 ± 2.5
33.	33.83	2580	Hexadecanoic acid, 2,3-dihydroxypropyl ester	12.2 ± 0.5	58.1 ± 3.4
34.	34.60	2634	Sucrose	19.9 ± 1.6	1.1 ± 0.2
35.	34.76	2635	Docosanoic acid	11.0 ± 0.7	28.7 ± 3.6
37.	36.67	2746	Tetracosan-1-ol	62.8 ± 2.8	1.6 ± 0.2
38.	37.26	2781	Octadecanoic acid, 2,3-dihydroxypropyl ester	3.3 ± 0.3	31.4 ± 3.1
39.	37.83	2811	Squalene	39.7 ± 1.5	247.6 ± 5.3
40.	39.98	2939	Hexacosanol	72.6 ± 2.3	8.4 ± 0.2
41.	40.63	2989	γ-Tocopherol	1.4 ± 0.1	ND
42.	41.45	3040	Hexacosanoic acid	6.8 ± 0.3	6.2 ± 0.9
43.	41.91	3061	Decanyl tetradecanoate	24.4 ± 1.2	4.6 ± 0.6
44.	42.95	3119	(+)-α-Tocopherol + Cholesterol	58.8 ± 4.2	55.8 ± 4.8
45.	43.08	3131	Octacosanol	28.0 ± 2.2	10.2 ± 0.7
46.	44.60	3229	Campesterol	195.2 ± 6.8	453.7 ± 4.3
47.	44.99	3261	Stigmasterol	128.0 ± 7.2	408.8 ± 6.7
48.	45.69	3321	Diploptene	147.3 ± 6.3	ND
49.	45.87	3324	β-Sitosterol	180.7 ± 2.8	153.8 ± 5.3
50.	46.02	3339	Isofucosterol	0.8 ± 0.1	44.6 ± 2.1
51.	46.93	3386	Cycloartenol	11.4 ± 2.1	ND
53.	49.06	3545	Phytylhexadecanoate	ND	6.8 ± 0.2

* Peak number as in Figure 1 and Figure 2; ** RT, retention time; *** RI_C_, retention index calculated for a temperature-programmed GC analysis at a constant heating rate by means of identified substance indices relative to a series of *N*-alkanes (C_8_–C_40_). Values represent the mean ± SD of three separate analyses. ND, not detected.

**Table 4:** Concentrations (mg/100 g dry weight) of substances from the chloroform extracts of the studied mosses according to the GC/MS analysis.

Identified Substance	AP	CD	DP	HS	PCC	PJ	PS	SF	SM	SR	ST
Tetradecanoic acid	14.9	4.9	16.9	14.7	13.5	0.4	28.6	4.2	3.1	63.2	12.8
Pentadecanoic acid	0.1	1.2	1.5	11.3	8.3	0.4	11.9	4.2	2.9	14.5	17.3
9-Hexadecenoic acid	ND	ND	ND	ND	ND	ND	11.7	ND	ND	ND	ND
*N*-Hexadecanoic acid	103.0	127.3	93.1	224.4	104.0	109.8	148.5	176.8	122.3	91.6	272.5
(*Z*)-3,7,11,15-Tetramethylhexadec-2-enoic acid	0.6	1.9	0.4	1.0	ND	ND	58.0	12.3	2.0	ND	4.3
Heptadecanoic acid	0.3	1.1	13.4	3.3	9.8	ND	148.5	11.4	0.7	4.0	12.0
9,12-Octadecadienoic acid (*Z*,*Z*)-	20.0	187.5	34.4	169.6	33.6	86.4	49.7	191.1	57.9	50.5	174.2
9,12,15-Octadecatrienoic acid	104.4	223.0	70.8	416.2	75.4	230.9	108.9	241.1	85.0	80.5	219.9
11-Octadecenoic acid	0.4	2.3	14.5	48.7	15.3	16.5	19.5	21.3	14.2	22.5	29.3
Octadecanoic acid	21.3	11.4	24.9	59.9	29.2	34.5	24.9	25.6	17.9	27.3	38.0
5,8,11,14-Eicosatetraenoic acid, (all-*Z*)-	1.8	89.0	36.6	238.3	22.2	12.9	65.9	44.1	17.7	31.6	23.9
5,8,11,14,17-Eicosapentaenoic acid	10.3	9.6	24.1	176.1	16.1	0.3	64.9	11.9	0.1	40.1	11.1
8,11,14-Eicosatrienoic acid	ND	24.9	15.1	42.2	17.0	0.2	18.2	2.7	0.5	ND	10.4
11,14-Eicosadienoic acid	ND	1.6	ND	21.1	ND	13.1	3.8	0.9	0.2	1.9	9.2
11-Eicosenoic acid	ND	ND	14.1	ND	11.3	23.1	10.5	4.7	0.3	ND	20.1
Eicosanoic acid	16.2	7.3	24.1	43.9	15.9	50.1	18.2	4.7	1.5	10.5	33.2
Docosanoic acid	17.7	18.0	21.9	49.7	26.4	57.4	23.1	27.1	23.0	36.8	32.6
Tricosanoic acid	16.8	ND	ND	ND	ND	14.2	ND	11.6	14.9	12.1	15.2
Pentacosanoic acid	0.4	0.3	ND	55.6	ND	30.2	26.1	10.3	13.4	39.4	15.5
Hexacosanoic acid	1.0	5.5	58.7	69.7	ND	40.0	23.6	28.1	47.7	81.0	48.6
Octacosanoic acid	0.5	3.4	7.1	61.6	398.2	50.7	13.4	2.1	21.1	6.3	17.3
1-Monopalmitoylglycerol	11.4	2.4	3.0	63.6	26.4	19.6	87.2	77.9	41.3	35.7	27.1
Octadecanoic acid 2,3-dihydroxypropyl ester	14.3	0.4	20.7	19.4	38.2	27.5	65.9	53.6	24.8	28.8	18.5
Neophytadiene	20.2	84.8	65.6	190.3	113.8	47.3	137.0	49.3	36.3	48.2	38.4
Phyta-1,3(*Z*)diene	1.0	10.1	5.0	20.4	ND	1.9	33.7	12.4	4.3	9.0	8.7
Phyta-1,3(*E*)diene	1.2	16.5	14.8	38.6	ND	1.3	54.5	18.4	7.5	12.7	11.1
Phytol	1.5	4.9	18.3	28.7	19.1	12.2	20.4	21.4	14.6	17.5	49.9
Squalene	174.5	42.9	64.7	117.6	43.6	206.3	111.3	14.1	19.7	37.3	25.7
1-Hexadecanol	ND	ND	11.0	ND	12.0	ND	ND	ND	0.2	ND	1.0
Hexadecane-1,2-diol	ND	ND	ND	22.2	ND	ND	ND	2.5	0.8	ND	ND
1-Octadecanol	0.2	1.4	2.1	ND	18.1	0.6	13.1	4.9	0.4	ND	ND
1-Docosanol	0.3	10.0	27.7	22.9	23.9	14.0	10.8	12.8	0.9	18.7	ND
1-Tricosanol	ND	ND	ND	19.4	ND	0.1	0.9	ND	ND	1.7	ND
1-Tetracosanol	1.5	16.6	21.7	38.0	30.7	28.2	25.6	25.0	19.0	76.9	24.1
1-Hexacosanol	20.4	28.1	49.8	55.6	83.0	3.6	26.1	34.0	30.2	117.0	44.1
1-Octacosanol	20.9	37.6	54.0	57.2	ND	5.2	98.1	50.2	22.4	4.4	49.0
1-Triacontanol	37.2	ND	ND	ND	ND	22.5	3.3	ND	ND	7.0	2.3
Cholesterol + α-Tocopherol	64.0	99.2	168.3	189.5	107.7	80.5	231.9	64.3	18.2	57.2	42.0
Desmosterol	1.0	ND	117.9	49.3	59.0	ND	102.3	ND	ND	63.2	ND
Ergosterol	ND	0.7	5.0	91.0	49.6	27.8	144.7	189.5	82.9	81.1	91.8
Campesterol	256.8	187.6	476.2	348.1	452.3	238.9	554.0	374.5	94.5	96.4	152.4
Stigmasterol	273.3	157.7	495.1	282.4	506.6	206.0	362.0	387.8	122.2	189.1	152.4
Ergosta-5,8-dien-3β-ol	ND	ND	ND	282.4	ND	ND	183.6	157.8	53.4	ND	ND
Ergost-7-en-3-ol	ND	ND	ND	ND	179.4	ND	ND	2.8	29.7	ND	18.3
β-Sitosterol	140.6	299.4	356.3	430.7	361.1	161.4	552.2	518.8	231.9	199.1	176.4
Pimaric acid	11.4	ND	ND	ND	26.6	ND	ND	12.2	0.3	ND	4.2
Isopimaric acid	ND	ND	16.3	22.9	ND	ND	24.8	15.7	ND	37.6	10.1
Dehydroabietic acid	12.5	14.6	16.9	61.5	20.0	13.0	71.0	11.9	ND	43.8	41.2
Abietic acid	ND	1.2	ND	25.9	5.1	ND	10.9	ND	ND	ND	3.8
Heneicosane	0.1	3.5	ND	19.3	ND	ND	ND	19.9	1.3	11.2	5.3
Tricosane	1.1	22.6	2.9	85.5	26.6	ND	ND	25.1	1.6	22.6	40.9
Pentacosane	11.1	20.9	13.4	53.7	29.7	13.9	23.0	22.4	13.8	19.9	17.9
Heptacosane	1.0	8.9	28.9	42.8	96.2	26.4	30.3	23.5	1.3	26.6	1.5
Nonacosane	20.8	9.7	60.4	77.9	21.3	16.2	39.3	11.5	0.7	35.3	14.5
Decanyl tetradecanoate	0.5	5.4	23.6	16.2	3.2	5.5	78.5	1.7	2.7	13.9	15.1
Phytylhexadecanoate	ND	ND	147.8	ND	ND	ND	83.2	38.0	ND	333.3	40.1
Cycloartenol	33.5	28.3	202.5	92.9	177.2	55.6	211.4	3.6	55.3	77.7	85.4
Ursolic acid	1.6	ND	ND	ND	ND	13.6	ND	3.5	598.2	143.7	188.9
Diploptene	ND	109.7	ND	280.3	86.1	ND	275.8	2.5	0.3	ND	ND
α-Amyrin	ND	18.5	ND	ND	ND	ND	ND	ND	ND	53.0	ND
Oleanolic acid	2.6	ND	ND	ND	ND	ND	ND	ND	ND	165.3	ND
Uvaol	ND	ND	ND	ND	ND	ND	ND	ND	ND	99.4	ND
Stigmasta-3,5-diene	2.5	5.2	11.2	22.5	ND	1.0	47.4	11.8	4.2	8.5	5.5

ND, not detected.

The analysis of moss extracts using the GC/MS method showed some substances that can be found only in some moss species. Due to the high complexity of the chemical composition of mosses, there are no single substances that are specific for moss species or genus and are not influenced by environmental impacts. The complexity of chemical composition hampers the use of secondary metabolite (for example, sterols, terpenoids, *etc.*) in chemotaxonomy. At this level of knowledge, relevant biomarkers stable with respect to environmental variability have not been identified. Nevertheless, the chemotaxonomic approach could be used to gain clearer understanding of moss taxonomy and chemistry. In previous studies, we have demonstrated that basic moss content (elemental composition, Py/GC/MS results, FTIR spectra) could be used for chemotaxonomic purposes [12]. In the case of extractable substances, the situation was more complex, and more detailed research with more moss species should be conducted to identify the genus characteristic substances, such as sphagnic acid and 9-hexadecenoic acid. Most substances of the triterpene group were specific only for some moss species; therefore, they could possibly be used for chemotaxonomic purposes. They could also influence the biological activity of moss extracts.

### 2.2. LC-TOF-MS Analysis of the Moss Ethanol Extract

In order to better characterize the possible sources of moss extract biological activity, the chemical composition of the ethanol extracts obtained by microwave extraction were analyzed using LC-TOF-MS (Table 5). Extraction with ethanol could help to isolate the substances with higher polarity, and groups of substances, like phenolics, carbohydrates and amino acids, could be identified. Using this extrahent and detection method, mainly phenolics, their glycosides and amino acids were analyzed in moss extracts. Like in the GC/MS analysis, and in ethanol extracts, too, the found variations between moss samples were significant, and no similarities that would be characteristic of the moss species or genus were found. Some remarkable substances with high biological activity, which have already been described in the literature, were found in the moss samples. High numbers of different phenolics were identified in the composition of some of the mosses for the first time, for example, 3ʹʹʹ-desoxydicranolomin, 5,6,7,8-tetrahydroxycoumarin-5-β-glucopyranoside, 7,8-dihydroxy-5-methoxycoumarin-7-β-sophoroside and others. The biologically-active substances found in the moss ethanol samples were matairesinol, apigenin, atraric acid, and others. In some samples, abscisic acid, a widely-known plant hormone, was also identified. We also identified in other moss species a substance that was previously supposed to be characteristic only for *Sphagnum* *mosses* (sphagnic acid). Noteworthy, in *Sphagnum rubellum* moss, some unique substances not characteristic for other tested mosses were found, such as hydroxyharmane and harmol propionic acid ester, which are derivatives of alkaloid harmol and have been found only in water moss at this time [1].

The total quantities of the main identified substance groups (Table 6) showed that the most abundant substance group in the moss chloroform extracts was fatty acids (329–1707 mg/100 g dry moss) and sterols (632–2130 mg/100 g dry moss). Overall, the main substituent groups in the moss extracts were amino acids (590–3266 mg/100 g dry moss) and fatty acids. In comparison to previous studies, many substances were not identified in moss *Hylocomium splendens*, such as α-pinene, limonene, pinocarvone, and others [15]. Nevertheless, some new substances for this moss species were identified, such as neophytadiene, phytol and squalene.

**Table 5:** Data of the LC-TOF-MS analysis of moss ethanol extracts.

No.	Formula	PC	RT	AP	CD	DP	HS	PCC	PJ	PS	SF	SM	SR	ST
v1	Atraric acid	3.0	27.3	14.6	44.8	5.9	4.4	14.9	23.8	74.3	2.0	4.3	5.3	7.9
v2	Benzyl benzoate	1.6	3.4	4.1	4.2	ND	6.9	5.4	8.8	12.6	7.5	13.2	ND	5.1
v3	4-Hydroxybenzoic acid	5.6	17.7	10.1	16.2	11.2	10.1	0.8	ND	9.4	7.4	11.5	11.8	4.7
v4	Methyl 4-hydroxybenzoate	2.5	2.8	7.6	8.8	4.1	6.5	0.5	10.1	7.7	1.5	18.9	2.3	7.2
v5	3-Methoxy-4-hydroxybenzoic acid	7.7	12.5	7.9	13.5	1.2	18.2	0.7	15.4	14.5	18.2	26.6	12.1	13.4
v6	4-*O-*Caffeoylquinic acid	ND	ND	ND	25.7	ND	5.0	ND	ND	5.3	7.9	1.9	15.3	11.6
v7	5-*O-*Caffeoylquinic acid	ND	ND	ND	25.7	ND	5.0	ND	ND	5.3	7.9	1.9	15.3	11.6
v8	Caffeic acid	6.4	3.2	41.8	2.3	ND	4.0	5.8	2.9	4.4	ND	ND	2.1	ND
v9	*p-*Coumaric acid	22.3	6.7	11.4	5.9	1.0	3.5	0.2	12.9	11.2	3.5	7.3	11.5	5.0
v10	Ferulic acid	0.8	2.7	21.3	3.3	1.2	1.1	19.1	2.7	3.0	3.7	5.6	1.5	3.9
v11	7,8-Dihydroxy-5-methoxycoumarin-7-β-sophoroside	63.2	144.1	37.1	25.0	58.0	164.9	95.9	117.3	163.8	65.7	32.8	105.3	106.5
v12	5,6,7,8-Tetrahydroxycoumarin-5-β-glucopyranoside	ND	ND	ND	ND	ND	ND	ND	ND	2.6	ND	ND	ND	ND
v13	Abscisic acid	4.3	5.2	ND	3.9	0.7	5.3	3.9	5.9	6.5	3.7	12.5	2.9	7.3
v14	Sphagnic acid	3.4	5.5	10.0	6.4	3.6	4.4	20.3	4.4	2.1	21.9	109.0	19.0	21.8
v15	5,8-Dihydroxy-7-methoxycoumarin 5-β-glucopyranoside	35.8	1.0	7.2	6.1	12.8	4.3	4.5	4.1	2.6	9.8	6.6	30.6	10.5
v16	Dimethyl phthalate	0.8	2.7	21.3	3.3	1.2	1.1	19.1	2.7	3.0	3.7	5.7	1.5	3.9
v17	Dimethyl terephthalate	0.8	2.7	21.3	3.3	1.2	1.1	19.1	2.7	3.0	3.7	5.7	1.5	3.9
v18	Ohioensin H	208.8	ND	0.8	6.1	5.3	4.1	6.6	5.6	2.5	10.3	11.7	7.4	7.6
V19	Harmol propionic acid ester	ND	ND	ND	ND	ND	ND	ND	ND	ND	ND	ND	0.6	ND
v20	7-Hydroxyharmane	ND	ND	ND	ND	ND	ND	ND	ND	ND	ND	ND	0.6	ND
v21	Apigenin	ND	69.0	69.5	71.6	6.0	36.4	ND	ND	21.7	ND	5.8	ND	ND
v22	2,3-Dihydro-5ʹ,3ʹʹʹ-dihydroxyamentoflavone	6.1	25.0	24.7	47.4	ND	9.3	ND	78.8	3.3	ND	ND	ND	ND
v23	2,3-Dihydrodicranolomin	6.1	25.0	24.7	47.4	ND	9.3	ND	78.8	3.3	ND	ND	ND	ND
v24	Communin A	20.5	ND	ND	3.5	ND	ND	ND	ND	2.2	ND	1.4	ND	ND
v25	2,3-Dihydro-5ʹ-hydroxyrobustaflavone	ND	0.8	ND	ND	ND	ND	3.7	ND	ND	ND	ND	ND	1.3
v26	3ʹʹʹ-Desoxydicranolomin	ND	114.8	102.7	ND	ND	14.7	ND	ND	17.3	ND	0.7	ND	ND
v27	3-Hydroxy-β-ionone	4.9	62.0	91.0	9.7	5.8	66.3	7.7	14.6	21.9	2.1	36.6	5.9	14.5
v28	*p*-Hydroxyacetophenone	1.2	31.2	32.3	16.3	0.0	18.4	12.0	ND	6.2	344.4	63.1	25.2	67.7
V29	3,5-dioxohexanoic acid	179.9	98.3	99.3	43.7	99.0	97.6	162.7	91.4	118.3	64.2	70.4	76.5	103.9
V30	4-hydroxybenzoic acid	5.5	17.7	10.1	17.3	11.2	10.1	0.8	ND	9.4	7.4	11.5	11.8	4.7
v31	Α-d-furanallulose	ND	ND	ND	ND	ND	ND	6.3	ND	1.3	0.8	1.6	1.0	ND
v32	Benzoic acid	11.8	5.1	0.0	5.2	ND	8.9	4.1	8.8	15.0	4.9	4.8	11.0	13.1
v33	Protocatechuic acid	6.3	11.3	18.4	18.5	5.2	15.9	12.2	10.6	9.5	8.2	13.0	8.7	10.9
v34	Vanillic acid	6.8	12.5	8.2	13.7	4.2	18.2	10.1	15.4	14.5	11.4	13.6	9.0	13.4
v35	Matairesinol	ND	ND	ND	4.4	0.4	ND	ND	ND	ND	ND	ND	ND	ND
v36	Glycine	7.8	31.2	18.4	20.6	16.2	35.3	31.0	20.5	14.1	12.4	19.0	19.2	23.3
v37	Alanine	200.5	145.4	166.0	84.4	297.0	82.6	154.8	60.0	94.4	78.1	407.8	139.8	137.2
v38	Serine	42.1	77.2	57.4	43.8	72.2	112.5	176.1	144.5	178.6	27.4	46.0	27.1	89.7
V39	Glutamic acid	69.8	765.3	709.9	262.5	75.7	1039.5	890.0	846.4	1134.6	135.6	77.7	10.8	587.8
v40	Proline	21.5	95.0	55.6	105.8	24.6	99.6	61.8	37.2	42.3	17.9	46.6	25.3	73.4
v41	Valine	397.6	253.7	208.4	188.9	290.0	255.1	373.5	443.5	188.6	84.3	176.2	53.4	298.9
v42	Threonine	51.4	51.8	62.3	27.7	43.2	81.7	105.7	72.9	73.0	26.1	98.0	12.1	99.0
v43	Leucine	54.5	45.0	278.4	74.9	47.4	57.2	81.0	68.7	121.0	55.3	448.3	15.1	176.0
v44	Asparagine	147.6	ND	ND	ND	ND	ND	541.1	0.0	ND	ND	589.6	0.9	ND
v45	Aspartic acid	194.0	269.0	276.6	42.3	52.4	419.9	507.9	216.8	427.3	49.1	67.5	15.9	186.7
v46	Glutamine	29.9	9.4	8.3	4.9	5.4	5.6	6.1	17.7	7.2	ND	9.8	ND	1.6
v47	Lysine	3.3	0.8	30.5	30.5	2.7	4.4	5.5	1.2	2.6	0.5	6.2	9.1	3.1
v48	Phenylalanine	103.4	69.1	519.1	124.6	32.6	108.6	171.7	45.4	89.9	22.5	355.7	13.3	56.3
V49	Arginine	5.1	30.4	15.5	11.5	3.7	53.6	25.4	84.3	107.0	59.1	95.9	4.0	9.7
v50	Tyrosine	36.2	2.4	25.1	6.4	13.3	21.2	26.7	16.4	13.3	3.3	15.9	3.6	8.6
v51	Tryptophan	75.4	20.7	378.7	159.6	11.6	35.6	107.4	45.9	42.4	18.5	161.1	27.2	38.3

ND, not detected.

**Table 6:** Substance groups (mg/100 g dry moss) found in the moss extracts using the GC/MS and LC-TOF-MS analysis.

Total Substance Group Amounts	AP	CD	DP	HS	PCC	PJ	PS	SF	SM	SR	ST
Fatty acids	329.5	719.9	471.3	1707.2	796.2	771.1	877.7	836.3	446.5	614.0	1017.4
Monoglycerols	46.1	2.8	23.7	166.6	123.0	56.1	283.5	161.2	86.2	106.5	63.9
Terpenoids	198.3	159.3	168.3	395.5	176.4	269.0	356.9	115.6	82.4	124.8	133.7
Alcohols	80.7	93.7	166.3	215.3	167.7	74.2	177.9	129.5	73.8	225.6	120.5
Sterols	735.7	744.6	1618.6	1673.4	1715.8	714.6	2130.6	1695.5	632.8	686.2	633.2
Diterpenes	23.9	15.8	33.1	110.2	51.7	13.0	106.7	39.8	0.3	81.4	59.3
Alkanes	34.1	65.6	105.6	279.3	173.8	56.5	92.6	102.4	18.7	115.6	80.1
Wax/Wax esters	0.5	5.4	171.4	16.2	3.2	5.5	161.7	39.6	2.7	347.2	55.2
Triterpenes	37.7	156.4	202.5	373.2	263.3	69.3	487.2	9.5	653.8	539.2	274.4
Steroids	2.5	5.2	11.2	22.5	ND	1.0	47.4	11.8	4.2	8.5	5.5
Polyphenols	651.9	711.2	704.6	509.3	252	559.3	440.9	521.8	580.3	631.6	504.3
Amino acids	1440.1	1866.4	2810.2	1188.4	988	2412.4	3265.7	2121.4	2536.3	590.1	2621.3

ND, not detected.

### 2.3. Evaluation of the Biological Activity in Moss Extracts

In order to evaluate the biological activity in moss extracts, two principal types of analysis were carried out: evaluation of antiproliferative activity on six cancer cell lines and antimicrobial activity on four stems.

All extracts demonstrate the ability to inhibit the development of bacteria (the inhibition zone diameter varied from 9 to 15 mm). In all moss extracts, antibacterial activity was found against *Bacillus cereus*. The highest activity (inhibition zone diameter 12 mm) against *Bacillus cereus* was found in the extracts from *Climacium dendroide* and *Polytrichum commune*, and the lowest activity in the extracts from *Hylocomium splendens* and *Sphagnum magellanicum* (inhibition zone diameter 9 mm). The highest activity (inhibition zone diameter 15 mm) was observed in the extract from *Polytrichum commune* against the *Staphylococcus aureus* bacteria. Some moss species showed antibacterial activity (inhibition zone diameter 10 mm) against *E.coli*: *Climacium dendroides*, *Ptilium crista-castrensis*, *Rhytidiadelphus triquetrus* and *Sphagnum magellanicum.* Extract of *Hylocomium splendens* did not show antimicrobial activity against *Escherichia coli*, which has been found in other studies [15]. *Hylocomium splendens* and *Pleurozium schreberi* also showed activity (inhibition zone diameter 12 mm) against *Pseudomonas aeruginosa.* Overall, there was antimicrobial activity present that could be potentially relevant for further research, especially in the case of *Polytrichum commune*, which showed the highest activity. None of the tested moss extracts show antifungal activity, which has been mentioned in other studies [15]. Unfortunately, no correlations between the total polyphenol or radical scavenging activity and the antimicrobial activity were observed. Therefore, chemical analysis cannot be regarded as the only means for determining the antimicrobial activity in mosses.

Biological activity in the extracts of mosses was tested as their ability to inhibit the proliferation of cancer cell lines. Six cancer cell lines were chosen based on the occurrence of cancer types (Table 7), and the same extracts for the antimicrobial activity tests were used. Only in some cases was no inhibition of cell proliferation detected, and none of the extracts showed a cytotoxic effect.

**Table 7:** Antiproliferative activity IC_50_ of the moss ethanol extract on 6 cancer cell lines, expressed as μg/mL of moss extract.

Species	Rat Glioma Cells (C6)	Human Epidermoid Carcinoma (A431)	Human Lung Carcinoma (A549)	Mouse Melanoma Cell Lines (B16-F10)	Human Breast Adenocarcinoma (MCF-7)	Human Colorectal Carcinoma (CaCo-2)
AP	51 ± 8	63 ± 8	100 ± 5	ND	100 ± 4	84 ± 4
CD	43 ± 7	64 ± 8	57 ± 8	98 ± 12	ND	66 ± 6
DP	3 ± 0.5	27 ± 4	57 ± 8	12 ± 2	67 ± 4	45 ± 4
PJ	68 ± 8	84 ± 9	24 ± 5	ND	>100	42 ± 5
PC	53 ± 5	23 ± 3	ND	24 ± 5	47 ± 6	24 ± 3
RT	86 ± 6	40 ± 5	98 ± 9	76 ± 8	ND	49 ± 4
PCC	23 ± 5	77 ± 8	32 ± 8	79 ± 8	>100	42 ± 5
PS	5 ± 0.4	28 ± 4	55 ± 7	39 ± 6	>100	61 ± 5
SF	27 ± 4	57 ± 4	>100	69 ± 9	ND	65 ± 3
ST	26 ± 3	62 ± 7	>100	ND	>100	41 ± 3
SM	0.9 ± 0.1	13 ± 2	44 ± 8	89 ± 9	ND	43 ± 4
SR	53 ± 5	72 ± 8	>100	ND	70 ± 5	42 ± 2

ND, not detected.

The best results were achieved when the extracts were tested on the rat glioma, human epidermoid carcinoma and human colorectal carcinoma cell lines. Some extracts (*Sphagnum magellanicum*, *Dicranum polysetum*, *Pleurozium schreberi*) showed particularly high inhibitory activity (0.9–5 μg/mL) on the rat glioma cells. Lower activity was observed on the human lung carcinoma, mouse melanoma and human breast adenocarcinoma cell lines. In the case of these cell lines, some extracts showed no activity or lower activity than in other cases.

### 2.4. Statistical Analysis

The next step of research was to analyze and understand a possible connection between the chemical content and the biological activity. Some methods of statistical analysis were tried, then choosing the linear correlation, followed by the pair analysis, using the Pearson correlation.

In order to evaluate the relations between the chemical composition and biological activity of moss extracts, statistical analyses were performed. Statistical processing was done on the data of cancer cell proliferation inhibition and the composition of moss ethanol extracts according to the LC-TOF-MS results.

The linear correlation analysis was performed in order to understand the effect of individual substances found in the moss ethanol extracts on the cancer cell proliferation inhibition activity for the rat glioma cells, human epidermoid carcinoma, human lung carcinoma, mouse melanoma cell lines, human breast adenocarcinoma and human colorectal carcinoma. The following correlation pairs were distinguished according to the analysis (Pearson correlation factors and significance are given in parentheses):

(1) rat glioma cells: 2,3-dihydro-5ʹ,3ʹʹʹ-dihydroxyamentoflavone and 2,3-dihydrodicranolomin (*r* = 0.587, *p* = 0.05);
(2) human lung carcinoma: ohioensin H (*r* = −0.579, *p* = 0.05); communin A (*r* = −0.610, *p* = 0.05);
(3) human colorectal carcinoma: 5,8-dihydroxy-7-methoxycoumarin-5-β-glucopyranoside and 5,8-dihydroxy-7-methoxycoumarin-5-β-glucopyranoside (*r* = −0.599, *p* = 0.05); apigenin (*r* = 0.751, *p* = 0.01); 3ʹʹʹ-desoxydicranolomin (*r* = 0.714, *p* = 0.01); 3-hydroxy-β-ionone (*r* = 0.665, *p* = 0.05).

**Figure 3:**
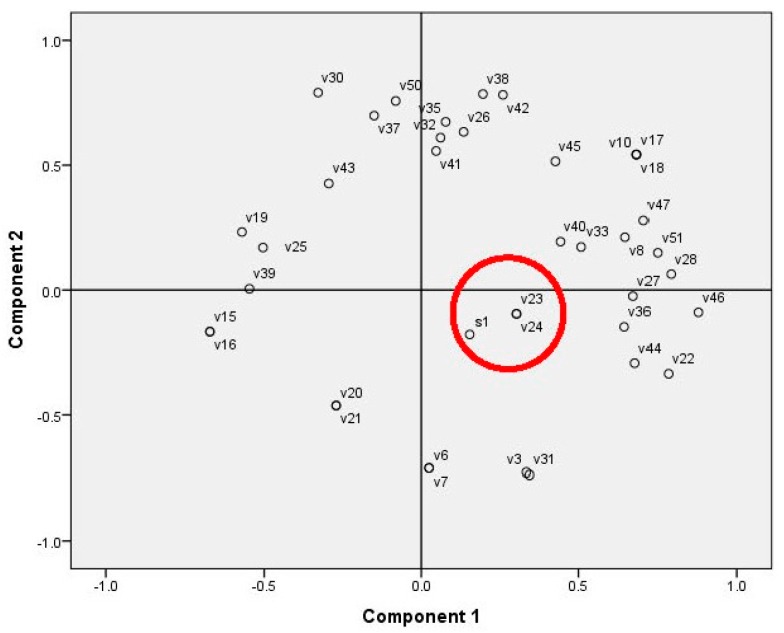
PCA on the rat glioma cell line proliferation inhibition and concentration of moss secondary metabolites (area marked with red circle: the glioma cell proliferation data (S1) and substance numbers (v1–v51) according to Table 5 were used as variables).

While a positive Pearson correlation factor indicated decreased proliferation activity, the negative values showed the specific substances that could be responsible for this effect. Strong associations with other groups of substances found in the moss ethanol extracts were not recognized according to the PCA (Figure 3), showing similar results to those of the correlation analysis.

**Figure 4:**
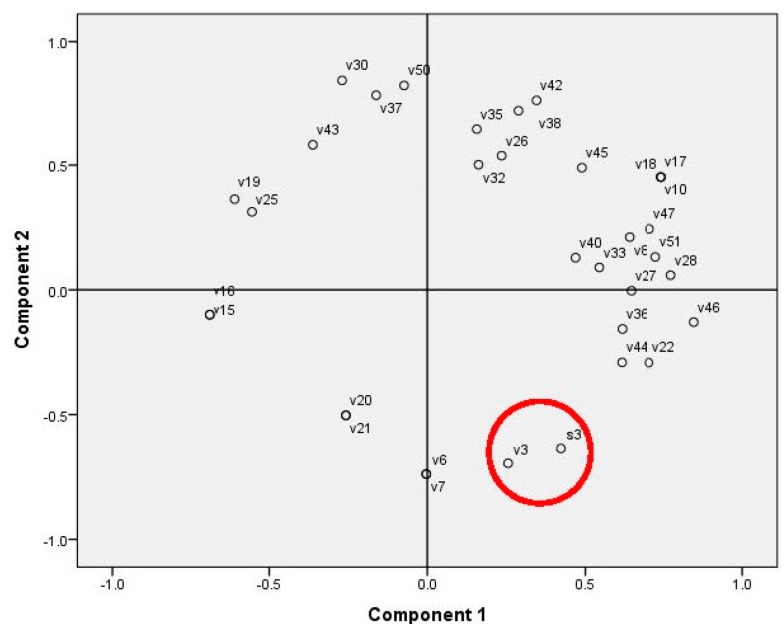
PCA on the cell line proliferation inhibition and concentration of moss secondary metabolites (area marked with red circle: the human lung carcinoma proliferation data (S3) and substance numbers (v1–v53) according to Table 5 were used as variables).

In the case of human epidermoid carcinoma cells, no relations to specific substances and components were found. Differently from the correlation analysis, PCA (Figure 4) showed that the human lung carcinoma cells were possibly enclosed together with 4-hydroxybenzoic acid. Axes 1 and 2 significantly explain 52% of the variation.

**Figure 5:**
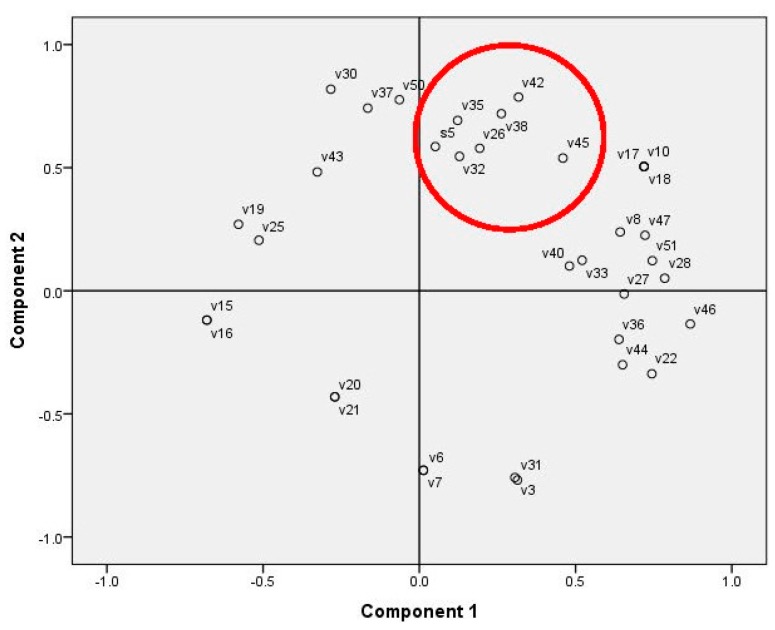
PCA on the cell line proliferation inhibition and concentration of moss secondary metabolites (area marked with red circle: the human breast adenocarcinoma proliferation data (S5) and substance numbers (v1–v53) according to Table 5 were used as variables).

The analysis of human breast adenocarcinoma cells, differently from the previous analysis, showed that the cell condition could be estimated together with the 2,3-dihydro-5ʹ-hydroxyrobustaflavone, Α-d-furanallulose, serine, threonine and aspartic acid concentrations (Figure 5).

The human colorectal carcinoma cells, according to the correlation analysis, showed highly similar variations with a wide spectrum of substances. According to PCA, the situation was quite similar, and the cells were located in one group with caffeic acid, ferulic acid, dimethyl phthalate, dimethyl terephthalate, apigenin, 3ʹʹʹ-desoxydicranolomin, 3-hydroxy-β-ionone and protocatechuic acid.

PCA can be used as a method of data analysis in order to determine the biological activity of moss extracts.

## 3. Experimental Section

### 3.1. Materials

A high variety of bryophyte species, including mosses, also the moss species used in this study, are common in Northern Europe [26,27]. The samples were collected in moist coniferous forests, moist deciduous forests and bogs in Latvia. The living parts of mosses were collected, then the samples were washed with distilled water to clean them from microorganisms; they were also cleaned from dirt, other possible moss species and needles. For further treatment, the plants’ top parts (2–5 cm) were taken, and then, the cleaned samples were stored at −20 °C. The moss species used and their specific growth environments are shown in Table 1. Vouchers of each bryophyte species specimen are stored at the Department of Environmental Science, University of Latvia.

All solvents were of analytical grade (Sigma-Aldrich, St. Louis, MO, USA). Internal standards (palmitic acid methyl ester, progesterone) were obtained from Sigma-Aldrich and used as solutions in chloroform in the concentrations indicated. *N*,*O*-Bis (trimethylsilyl) trifluoroacetamide + trimethylchlorosilane (Fluka analytical), acetonitrile, formic acid and gallic acid were purchased from Sigma-Aldrich. The used deionized water (18.2 MΩ) was prepared by a Milli-Q water purification system from Millipore (Billerica, MA, USA). The stock solution of gallic acid with a concentration of 1.0 mg/mL was prepared by dissolving 50 mg of gallic acid in 50 mL 50% aqueous acetonitrile. Working solutions of all of the standards were prepared immediately before analyses by diluting the stock solution with 50% aqueous acetonitrile to attain the required concentrations for calibration measurements (0.5, 0.25, 0.05, 0.025, 0.005 and 0.0001 mg/mL).

### 3.2. Extraction and Sample Preparation Procedures

#### 3.2.1. Extraction with Ethanol

The samples were dried at +40 °C in an oven until constant mass. The dry moss samples were ground in a mill, and 1 g of bryophyte samples was weighed into 100-mL Teflon extraction vessels with screw caps, adding 50 mL of 60% ethanol solution. The vessels were sealed using a Milestone Twister (Milestone, Sorisole, Italy). Extraction was performed using a Milestone Ethos One microwave oven (Milestone), at 150 °C, with 1500 W of power. The extraction took 40 min: 10 min to reach the chosen temperature, 20 min for steady extraction at the set temperature and 10 min for the oven to cool down. After the extraction had been completed, the samples in the extraction vessels were placed at room temperature and held still for approximately 1 h for the extract to cool down. All prepared extracts were filtered and stored at 4 °C for up to 1 month, until the analysis.

#### 3.2.2. Extraction with Chloroform

Dry (dried at 40 °C until constant weight) moss samples were ground in a mill, and 10 g of the samples were weighed into 1-L bottles with screw caps, adding 300 mL of chloroform. After that, the samples were exposed to ultrasound (100 W) in an ultrasound bath (Cole Parmer, Vernon Hills, IL, USA) for 40 min. The temperature was kept constant at +40 °C by regularly adding cold water. The bottles were then shaken in a shaker for 24 h at 140 rpm. After 24 h 40 min, treatment with ultrasound was repeated. Then, the extracts were filtered and concentrated on a rotation evaporator.

#### 3.2.3. Derivatization of Substances in Chloroform Extracts

Twenty milligrams of the moss extract dry weight were weighed in a glass vessel. The dry residue was dissolved in 1.4 mL of acetonitrile, and then, 0.5 mL of internal standard (methylheptadecanoic acid and progesterone) and 0.1 mL of BSTFA reagent were added. Next, the vessel was tightly sealed and heated at +60 °C for one hour. In order to ensure that all BSTFA had reacted, 0.01 mL of methanol was added to the mixture. After cooling down at room temperature, the glass vessel with the mixture was used for further GC/MS analysis.

### 3.3. Analytical Methods

#### 3.3.1. Determination of Total Polyphenol Concentration

For determining the total amount of polyphenols, the Folin–Ciocalteu reagent was used [28]. Before analysis, the bryophyte extracts were kept at room temperature for ~1 h. One milliliter of the moss extract was added to a test tube, and 5 mL of 10% Folin–Ciocalteu reagent (Aldrich) were added. After 5 min, 4 mL of 7.5% sodium carbonate (Aldrich) were added. The test tube was then shaken thoroughly and kept in a dark place at room temperature for 2 h. Absorption was then measured in a quartz cuvette (d = 1 cm) on a spectrophotometer (Hach-Lange DR 2800, Düsseldorf, Germany) at a 725-nm wavelength. The results were calculated using a standard curve, which is expressed as mg of gallic acid/100 g (GE/100 g) dry matter [28,29]. Three parallel measurements were carried out.

#### 3.3.2. Analysis of the Total Amount Carbohydrates

Before analysis, the moss samples were kept at room temperature for ~1 h. Zero-point-one milliliters of the moss extract were added to a test tube and diluted to 1 mL with distilled water. Following that, 1 mL of 5% phenol (Aldrich) solution was added, also immediately adding 5 mL of concentrated sulfuric acid (Aldrich). In 10 min, the test tubes with the samples were carefully shaken and then left for 20 min at room temperature. Finally, the absorption was measured with a spectrophotometer (Hach-Lange DR 2800) at 490 nm. Three parallel measurements were carried out. The amount of carbohydrates was determined with a calibration curve using glucose solution as a standard [30].

#### 3.3.3. Determination of Radical Scavenging Activity Using DPPH

Before analysis, the moss samples were kept at room temperature for ~1 h. Zero-point-three milliliters of the moss extract were added to a test tube and mixed with 3.6 mL of 4% solution in 96% ethanol 2,2-diphenyl-1-picrylhydrazyl (DPPH) (Aldrich). The mixture was incubated for 20 min in a dark place at room temperature. The absorption was measured in a quartz cuvette (d = 1 cm) with a spectrophotometer (Hach-Lange DR 2800) at a 517-nm wavelength. Three parallel measurements were carried out. The radical scavenging activity is expressed as mg of gallic acid/100 g (GE/100 g) dry matter.

#### 3.3.4. Analysis of Lipid Extracts by Gas Chromatography-Mass Spectrometry

The GC-MS instrumentation consisted of a Clаrus 680 chromatograph and a Clаrus SQ 8 C mass spectrometer. The separations were performed on an Еlitе-5ms (5% phenyl/95% methyl polysiloxane) capillary column (30 m × 0.25 mm i.d., 0.25 µm film thickness). Helium (99.9999%) was used as the carrier gas at an initial flow rate of 2.0 mL/min for 2 min, after that being held constant at 1.0 mL with a split flow rate of 10.0 mL/min. The column temperature was maintained at 75 °C for 2 min, then programmed from 75–130 °C at 20 °C 1/min and from 130–310 °C at 4 °C 1/min and, finally, held at 310 °C for 12 min, with the total run time of 59.25 min. The sample of 1.0 µL was injected in a split mode injector (4/1) with an autosampler. The mass spectrometer was operated in the positive electron impact (EL+) mode at 70-eV ionization energy and scanned from 42–650 *m*/*z* with a cycle time of 0.5 s. The multiplier was operated at 1700 V. The column injector and transfer line temperatures were set at 290 °C and 250 °C respectively, and the ion source temperature was 230 °C. The retention time (Rt, min) and MS fragmentation patterns of the known compounds were compared to the literature and NIST database. All peaks were quantified by the peak area.

#### 3.3.5. Analysis of the Ethanol Extracts by Ultra-Performance Liquid Chromatography Coupled with Time-of-Flight Mass Spectrometry

Chromatographic analyses were performed on a modular UPLC system Agilent 1290 Infinity series (Agilent Technologies, Frankfurt am Main, Germany). LC separations were achieved by using an X Bridge C8 3.5 µm, 2.1 × 150 mm (Waters, MA, USA) column with a mobile phase composed of 0.1% formic acid (Solvent A) and acetonitrile (Solvent B) in the following linear gradient mode: initial: 2% A, 98% B 0–2 min; 95% A, 5% B 2–30 min; 95% A, 5% B 30–50 min; 2% A, 98% B 50–52 min. The flow rate was maintained at 0.3 mL/min. The injection volume was 10 μL. The UV detection of compounds was monitored using a diode array detector (Waters) at 210, 254, 280 and 330 nm.

The high resolution mass spectra (HRMS) were taken on an Agilent 6230 TOF LC/MS system(Agilent Technologies) with electrospray ionization (ESI). The source parameters were: positive ionization mode, drying gas flow 10 L/min and temperature 325 °C, fragmentor ionization 100 V. The internal reference masses of 121.050873 *m*/*z* and 922.00979800 *m*/*z* (G1969-85001 ES-TOF Reference Mass Solution Kit, Agilent Technologies & Supelco, Frankfurt am Main, Germany) were used for all analyses of the samples. The experimental data were handled using the MassHunter Version B05.00 software (Agilent Technologies). The ethanol extracts of moss were filtered through 0.45-μm filters (Nonpyrogenic Sterile-R, Sarstedt, Hemer, Germany) to remove solid particles and mechanical admixtures. The filtered samples were diluted with acetonitrile and injected into the LC system. The 0.75 μL of filtered samples were diluted with 0.75 μL of acetonitrile and injected into the LC system. The injections were performed in duplicate.

The calibration curve of standard solutions was constructed by plotting the ratio of the average chromatographic peak area and mass concentration of gallic acid. According to the reflected data, the regression equation of the trend line was calculated. Standard solutions were injected in triplicate, and the corresponding peak areas were recorded. The relative standard deviation was determined to be less than 1%. The obtained calibration curve showed the linearity of the correlation coefficient (*R*^2^) in the concentration range of 0.993.

#### 3.3.6. Cytotoxicity Testing of Moss Extracts

The following cell lines obtained from ATCC and European ECACC collections were used for testing: rat glioma cells (C6), human epidermoid carcinoma (A431), human lung carcinoma (A549), mouse melanoma cell lines (B16-F10), human breast adenocarcinoma (MCF-7) and human colorectal carcinoma (CaCo-2). The cytotoxicity of the extracts was tested on the monolayer cultures C6, A431, MCF-7 and CaCo-2. The cell lines A431, MCF-7 and CaCo-2 were cultivated in DMEM media with 10% fetal bovine serum, 2 mM glutamine and 1% amino acids without antibiotics. The C6 cells were cultivated in F12HAM media, containing 10% fetal bovine serum and 2 mM glutamine. Cells at a concentration of 5 × 10^4^ cells/mL (A431, MCF-7, Caco-2 cell lines) and 4 × 10^4^ cells/mL for the C6 cell line were sown in a 96-well plate and cultivated for 24 h at 37 °C, 5% CO_2_ atmosphere. Then, the cell extracts were added at different concentrations and cultivated for another 72 h. The living cells were determined by analyzing the activity of mitochondrial enzymes after staining with methylthiazolyldiphenyl-tetrazolium bromide (Sigma) (MTT). After the cultivation, the MTT solution (the final concentration of 2 mg/mL in HBSS buffer) was added and cultivated for an additional 3 h at 37 °C, 5% CO_2_. Then, the color was extracted with 0.2 mL DMSO. The optical density of DMSO extract (in proportion to the quantity of living cells) was measured at λ = 540 nm (Multiskan GO). The IC_50_ (μg/mL) was calculated with GraphPad Prism^®^.

#### 3.3.7. Antimicrobial Activity of Moss Extracts

The antimicrobial activity of moss ethanol extract was determined with the agar well diffusion method [31]. The microorganisms used in the experiment were obtained from the Microbial Strain Collection of Latvia. The antimicrobial activity was tested using these bacteria: *Bacillus cereus*, *Pseudomonas aeruginosa*, *Staphylococcus aureus* and *Escherichia coli.* The antimicrobial activity testing was performed on Mueller–Hinton agar. Fresh bacteria material with approximately 10^6^ colony-forming units/mL of tested organism was prepared. Sixty percent aqueous ethanol moss extracts of 70 μL of each sample and control (60% ethanol) were applied into 7.0 mm-diameter wells. Subsequently, the plates were incubated at 37 °C for 24 h. After the incubation, the inhibition zone around the well was measured in mm and used to express the antimicrobial activity. Each sample was tested in triplicate.

#### 3.3.8. Statistical Analysis

The statistical analysis was conducted using the IBM SPSS (version 22, IBM, Chicago, IL, USA) software. The linear correlation analysis was performed in order to understand the variation of the individual substances in the moss ethanol extract within the cancer cell lines. The principal component analysis (PCA) results were obtained by Varimax rotation, and Kaiser normalization was used, as well.

## 4. Conclusions

The relatively poorly studied mosses common in northern regions of Europe have high numbers of secondary metabolites in their composition. The analysis of the extracts demonstrates the presence of high numbers of different substances, and several of them have been identified for the first time in these moss species. Furthermore, high numbers of polyphenols and amino acids have also been identified in the moss extracts. The main groups of primary and secondary metabolites in the moss extracts are amino acids, fatty acids and sterols. The tested moss extracts do show antimicrobial activity on several bacteria strains (*B. cereus*, *E. coli*, *S. aureus*, *P. aeruginosa*). The most potent extract with the highest antimicrobial activity was found to be from the moss *Polytrichum commune*. The results of the multivariate analysis of the moss secondary and primary metabolite chemical composition and biological activity data show a variety of substances, which could be isolated and further tested to confirm the results and understand the mechanism of how they cause decreased proliferation activity in cancer cell lines.
